# Surgical site infections and sepsis in gynecological surgery

**DOI:** 10.1002/ijgo.70356

**Published:** 2025-07-09

**Authors:** Cristina Taliento, Gennaro Scutiero, Carmelia Milano, Mattia Grasso, Francesca Nitti, Orsola Brasile, Ruby Martinello, Giulia Bernardi, Stefano Restaino, Martina Arcieri, Matteo Pavone, Nicolò Bizzarri, Giuseppe Vizzielli, Pantaleo Greco

**Affiliations:** ^1^ Department of Medical Sciences, Institute of Obstetrics and Gynecology University of Ferrara Ferrara Italy; ^2^ Department of Development and Regeneration KU Leuven Leuven Belgium; ^3^ Department of Maternal and Child Health, Obstetrics and Gynecology Clinic, Ospedale Santa Maria Della Misericordia Azienda Sanitaria Universitaria Friuli Centrale Udine Italy; ^4^ Dipartimento per la Salute Della Donna e del Bambino e Della Salute Pubblica, UOC Ginecologia Oncologica Fondazione Policlinico Universitario A. Gemelli IRCCS Rome Italy; ^5^ ICube, Laboratory of Engineering, Computer Science and Imaging, Department of Robotics, Imaging, Teledetection and Healthcare Technologies University of Strasbourg, CNRS, UMR Strasbourg France; ^6^ IRCAD Strasbourg France; ^7^ Department of Medicine (DMED) University of Udine Udine Italy

**Keywords:** ERAS, gynecology, sepsis, SSI, surgical site infections

## Abstract

Despite advancements in infection control, surgical site infections (SSIs) and postoperative sepsis remain significant challenges in gynecological surgery, contributing to increased morbidity, mortality, and healthcare costs. In low‐income countries, circulatory failure due to septic shock leads to most deaths after surgery, with sepsis accounting for almost two‐thirds. This review provides a comprehensive update on the global incidence, risk factors, pathogenesis, and preventive strategies for sepsis and SSIs in gynecological procedures. Utilizing a search of PubMed and Web of Science, recent evidence highlights the complex interplay of microbial, patient‐specific, and procedural factors influencing infection risk. Key findings underscore the importance of modifiable risk factors, including diabetes, obesity, and anemia, as well as the role of the vaginal microbiota in postoperative infections. Enhanced Recovery After Surgery (ERAS) protocols, including targeted antibiotic prophylaxis, optimal skin preparation, and perioperative glycemic control, have shown efficacy in reducing SSIs and associated complications. This review emphasizes the critical need for tailored infection prevention strategies to mitigate the burden of sepsis in gynecological practice.

## 1 INTRODUCTION

By the mid‐1960s, advances in infection prevention and treatment improved surgical outcomes in various fields of medicine, including gynecology.^1^ New practices to improve safety have gradually spread through hospitals, leading to a decrease in serious infections and “failure to rescue”, which occurs when healthcare providers fail to recognize or respond to early signs of patient deterioration.^2^, ^3^ Despite these improvements, surgical site infections (SSIs)—defined as infections involving the superficial or deep incision, or an organ/space, occurring within 30 days post‐surgery—still account for 20% of all hospital‐acquired infections.^4^ SSIs are independent risk factors for sepsis and contribute substantially to postoperative complications, increased healthcare costs, and prolonged hospital stays.^5^ Sepsis and septic shock are life‐threatening conditions resulting from a dysregulated immune response to infections, leading to tissue and organ injuries that can culminate in death.^6^ In surgical practice, sepsis represents a significant cause of morbidity and mortality worldwide, accounting for approximately one‐third of all sepsis cases.^7^

The introduction of Enhanced Recovery After Surgery (ERAS) protocols in gynecologic oncology has significantly impacted the prevention of postoperative infections.^8^ ERAS integrates evidence‐based strategies, including targeted antimicrobial prophylaxis, optimal skin preparation, perioperative glycemic control, and avoidance of hypothermia.^9^ Studies have demonstrated that the implementation of ERAS protocols is associated with a substantial reduction in SSIs and sepsis‐related complications in both gynecologic oncology and general gynecology.^10^, ^11^ Moreover, this approach has proven to be an effective model for improving postoperative outcomes and mitigating the clinical and economic burden of infections.^12^

This review provides an updated analysis on the incidence of sepsis from SSIs, exploring its biochemistry and implications in gynecologic surgery, with a focus on preventive strategies such as those outlined in ERAS protocols.

## 2 MATERIALS AND METHODS

The electronic search databases used were PubMed and Web of Science in March and April 2024. We selected papers using the following keywords: “sepsis,” “sepsis in gynecological surgery,” “post‐operative surgical site infections,” “complications of surgery,” “treatment of surgical site infection.” We provide an updated overview of the definition, risk factors, pathology, diagnosis, and recommendations based on the latest ERAS guidelines.

## 3 EPIDEMIOLOGY OF SEPSIS AND SURGICAL SITE INFECTIONS

Sepsis is a clinical condition associated with a high mortality rate and a long‐term morbidity. It is considered a public health issue with substantial economic consequences. In 2017, The World Health Assembly and WHO recognized sepsis a global health priority and adopted a resolution to enhance its prevention, diagnosis, and management.^13^

In the same year, an estimated 48.9 million new cases of sepsis were recorded worldwide, with approximately 11 million sepsis‐related deaths, accounting for 19.7% of all global deaths. From 1990 to 2017, the age‐standardized incidence of sepsis decreased by 37% and mortality decreased by 52.8%.^14^ In the United States, in a retrospective study including 409 hospitals from 2009 to 2014, sepsis was present in 35% of all hospitalizations that culminated in death.^15^ A similar mortality rate was observed in European hospitals.^16^

The SOAP study showed that the most common site of infection in sepsis cases was the lungs (68%), followed by the abdomen (22%), blood (20%), and urinary tract (14%). Methicillin‐resistant *Staphylococcus aureus* was isolated from 14% of cultures, and the most common Gram‐negative organisms were *Pseudomonas* (14%) and *Escherichia coli* (13%).^16^

In high‐income countries (HICs) such as the United States and western Europe, the incidence of postoperative sepsis ranges between 1% and 3%. An analysis of a total of 1 276 451 surgery discharges from 1990 to 2006 in the USA showed that 2.9% of all surgical procedures were complicated by sepsis, with a higher risk of developing sepsis after non‐ elective procedures (4.2% and 1.1%, respectively; *P* < 0.0002).^17^

SSIs represent 20% of all hospital‐acquired infections and are independent predictors of postoperative sepsis.^4^

However, the incidence rates of sepsis and SSIs are significantly higher in low‐ and middle‐Income countries (LMICs).^18^ A secondary analysis of the FALCON trial, which included 5558 patients who underwent abdominal surgery in 54 hospitals across seven LMICs, showed that 74% of postoperative deaths were due to circulatory system failure, with 57% of these deaths attributed to sepsis.^19^ Furthermore, differences in the incidence of sepsis‐complicated infections after major gynecologic surgery have been reported to be greater in low‐volume hospitals than in high‐volume hospitals.^20^

## 4 PATHOGENESIS

In operative gynecology, infections that occur during the first postoperative day are usually caused by Gram‐positive cocci or, occasionally, by facultative Gram‐negative bacteria. Infections that occur after the second postoperative day are more likely to have an anaerobic component. Pelvic infections are polymicrobial in nature: 20% are caused by anaerobic Gram‐positive cocci, 20% by Gram‐negative bacilli, and 60% by anaerobic species.^21^, ^22^

The pathogenesis of sepsis is extremely complex, initiated by external or internal insults to cells that activate a cascade of immunohistochemical reactions, ultimately leading to altered tissue and organ function.^6^

The underlying pathogenetic mechanism involves dysregulation towards proinflammatory pathways. The immune system is activated by pathogen ligand and recognition receptors (PRRs) on the cell surface or in the cytosol, including nucleotide‐binding oligomerization domain (NOD)‐like receptors and RIG‐I‐like receptors. These receptors detect DAMPS (endogenous molecules released from damaged host cells, including ATP, mitochondrial DNA, or HMGB1) and PAMPS (pathogen‐associated molecular patterns), which include components of bacterial, fungal, and viral pathogens. This activation leads to the cascade production of inflammatory cytokines, such as interleukin‐1 (IL‐1), IL‐6, tumor necrosis factor (TNF), interferon (IFN), regulatory factor 7 (IRF7), and adapter protein 1 (AP‐1).^23^ Some pattern recognition receptors, mainly NOD‐like receptors, can assemble into molecular complexes called inflammasomes. These inflammasomes are involved in the release of the cytokines IL‐1β and IL‐18, and can trigger a form of programmed cell death known as pyroptosis, which is mediated by caspases and characterized by rapid mediated rupture of the plasma membrane.^24^ In patients who develop sepsis, the response is exaggerated, or “hyperinflammatory”, leading to the release of reactive oxygen species (ROS), which can damage cellular proteins, lipids, and DNA, impair mitochondrial function, and activate the complement system. This further increases ROS generation, granulocyte enzyme release, endothelial permeability, and tissue factor expression and can cause adrenal medullary cell death and organ damage.

One critical consequence of sepsis is disseminated intravascular coagulation, which is associated with organ dysfunction, bleeding, and high‐risk mortality. In the lungs, endothelial dysfunction can result in acute respiratory distress syndrome (ARDS). In the bowel, epithelium dysfunction can cause bacteria translocation, activation of pancreatic enzymes and subsequent self‐digestion (pancreatitis). Additionally, sepsis impairs bilirubin clearance in hepatocytes, causing cholestasis and ultimately liver failure.^25^ Acute kidney insufficiency is a common complication of sepsis, significantly increasing the risk of death. Encephalopathy is an early and frequent clinical manifestation in severe sepsis, ranging from mild cognitive impairment to deep coma.^26^

## 5 RISK FACTORS OF SURGICAL SITE INFECTIONS AND SEPSIS

Existing evidence has shown that risk factors for SSI include age, obesity, diabetes, tobacco smoking, systemic steroid use, anemia, blood transfusion, surgical site irradiation, malnutrition, general anesthesia, and prolonged preoperative stay (Table 1).^27^, ^28^, ^29^ Many of these risk factors are modifiable, and efforts to address such factors may decrease the risk of postoperative infection. In the United States, a cross‐sectional analysis performed by Lake et al. found that variables significantly associated with cellulitis after hysterectomy included: diabetes (*P* < 0.001), obesity, (*P* < 0.001), ascites (*P* < 0.01), weight loss (*P* = 0.02), cancer (*P* < 0.001), ASA class 3 or greater (*P* < 0.001), general anesthesia (*P* = 0.001) and an operative time >75th percentile (*P* < 0.001).^30^

**TABLE 1:** Risk factors for postoperative infections.

Risk factors of surgical infections
Hospitalization
Age
Blood transfusion
General anesthesia
Diabetes
Obesity
Smoking
Radiation therapy
Anemia
Steroid drug

Similarly, in a prospective multicenter cohort study, Thelwall et al. reported that obesity significantly increases the risk of postsurgical infection across various surgical categories. The mechanism is likely multifactorial, involving impaired tissue oxygenation, which slows the healing process, as well as the difficulty in achieving adequate tissue levels of prophylactic antibiotics.^28^

Moreover, several epidemiological studies have shown that diabetes is another risk factor. The association between diabetes mellitus and SSIs is related to the negative effect of hyperglycemia on immune function, especially on chemotaxis, phagocytosis, and granulocyte adhesion. Diabetic patients may also be predisposed to infections due to vascular insufficiency, peripheral sensory neuropathy, autonomic neuropathy, colonization of the skin and mucosa by pathogens such as *Staphylococcus aureus* and *Candida* species. In a meta‐analysis of four randomized controlled trials (RCTs) and six cohort studies, Boreland et al. showed that glycemic control using continuous insulin infusion (maintaining glucose levels ≤200 mg/dL) significantly lowered SSI rates following cardiac surgery compared with subcutaneous insulin.^29^ In a gynecological setting, a retrospective analysis of 56 640 laparoscopic hysterectomies from the American College of Surgeons National Surgical Quality Improvement Program database, showed that insulin‐dependent diabetes mellitus patients had a higher incidence of superficial surgical site wound infection (*P* = 0.049) and sepsis (*P* = 0.043) as compared with non‐diabetics.^31^

Preoperative anemia is another factor associated with increased sepsis. Richards et al. reported that preoperative anemia in gynecological surgery was independently associated with increased 30‐day mortality and a higher riskof sepsis (odds ratio [OR] 2.64, 95% confidence interval [CI] 1.81–3.86).^32^ In patients undergoing minimally invasive hysterectomy for benign indications, Tyan et al. found that perioperative allogeneic blood transfusion remained significantly associated with infectious wound events (*P* < 0.001), and sepsis (*P* < 0.001).^33^ Intraoperative factors also play a role. In a retrospective study including 303 834 women who underwent cesarean delivery, general anesthesia was associated with a higher incidence of SSIs compared with neuraxial block, with a OR of 3.7.^34^ This finding is consistent across other surgical fields. A recent meta‐analysis found that regional anesthesia was associated with a lower incidence of sepsis in patients who underwent vascular surgery.^35^ In another study, patients undergoing primary hip and knee arthroplasty experienced a reduced risk of infections with neuraxial anesthesia compared with general anesthesia.^36^

Furthermore, for patients undergoing gynecological oncology operations, a single‐institution study reported that pelvic lymphadenectomy, para‐aortic lymphadenectomy, splenectomy, bowel resection, or pelvic exenteration for surgical treatment of gynecologic malignancies are associated with increased risk of deep superficial and organ space SSIs.^37^

### 5.1 The impact of genital tract microbiota on post‐surgical infections

The vaginal microbiome is a complex and dynamic micro‐ecosystem that undergoes continuous fluctuations throughout the menstrual cycle and across a woman's life span. A healthy vaginal microbiome is typically dominated by the presence of *Lactobacillus* species, which produce various antimicrobial compounds—such as hydrogen peroxide and lactic acid—that help to maintain an acidic vaginal pH. This acidity represents the first line of defense against pathogenic microorganisms.^38^, ^39^ Several factors can alter the composition of the vaginal microbiome, including antibiotics, smoking, vaginal showers, pregnancy, lactation, and recent bacterial infections.^26^ The most common bacteria include *Lactobacillus* species, *Gardnerella vaginalis*, *Staphylococcus epidermidis*, *Corynebacterium* species, *Enterococcus faecalis*, *Streptococcus* species, and Enterobacteriaceae.

Furthermore, the introduction of new molecular engineering techniques has revealed a diverse community of bacterial species colonizing the uterine cavity. Michell et al. carried out a quantitative analysis of molecular DNA on uterine swabs and identified multiple microorganisms that colonize the uterine cavity, including *Atopobium vaginae*, *Prevotella*, *Lactobacillus crispatus*, *Lactobacillus iners*, and *G. vaginalis*.^40^ The recent introduction of cloning and sequencing of the 16S rRNA sequence has allowed further microorganisms to be isolated from the uterine cavity. Franasiak et al. in 2016 characterized the microbiome of 33 women from material collected during embryo transfer (ET) procedures for assisted reproductive technologies, with *Lactobacillus* and *Flavobacterium* being the most frequently isolated genera.^41^ In 2017, Tao et al. analyzed ET samples from 70 women, identifying *Lactobacillus*, followed by *Corynebacterium*, *Staphylococcus*, *Streptococcus*, and *Bifidobacterium* as the most common bacteria.^42^

The microbiota plays a key role in the development of SSIs in gynecological procedures. In most cases, these infections are caused by the colonization of the cutaneous and vaginal endogenous flora. These organisms are mainly Gram‐positive aerobic cocci (staphylococci) but may also include fecal flora bacteria (aerobic Gram‐negative and anaerobic bacteria) in cases where the incision is close to the perineum.^43^, ^44^

During gynecological surgery, vaginal or cervical flora can invade upward structures of the upper genital tract, such as endometrium, vaginal cuff, and parametrial tissue. This is supported by findings that the bacteria most frequently isolated in post‐hysterectomy cellulitis—such as *G. vaginalis*—are common components of the vaginal flora.^45^

The association between bacterial vaginosis (BV) and post‐surgical infectious complications is still not completely understood. However, evidence suggests there is a significant correlation. Soper et al. found that among 134 patients undergoing abdominal hysterectomy, those with preoperative BV had a significantly higher risk of vaginal abscesses and cellulitis compared with those without BV (34% vs. 11%).^45^ Larrson et al. observed a relative risk of 4.4 for developing postoperative infections in women with BV undergoing abdominal hysterectomy for benign causes.^46^ Similarly, a large nation‐wide Swedish study confirmed that BV was associated with an increased risk of wound‐cuff and/or deep infection following hysterectomy.^47^ These findings suggest that perioperative screening and treatment of BV could be beneficial in reducing SSI rates in patients scheduled for hysterectomy.^44^

## 6 POSTOPERATIVE INFECTIONS AFTER GYNECOLOGICAL SURGERY

Common infections observed after gynecological surgery include urinary tract infections (UTIs), particularly catheter‐associated UTI, and SSIs. SSIs occur in up to 20%–30% of gynecologic oncology patients undergoing a laparotomy.^48^ Additionally, colon surgery, which is involved in up to 45% of gynecologic oncology debulking procedures, is recognized as one of the surgical procedures with the highest risk of SSIs, associated with a 2.67‐fold increased risk.^49^, ^50^

Table 2 summarizes common infectious conditions following gynecological surgery.

**TABLE 2:** Infections after gynecological surgery.

Infection	Definition	Incidence	Type of surgery	Timing	Signs and symptoms	Diagnosis
Urinary tract infections	Urinary tract, including bladder, urethra or kidneys	0%–13% after hys	Hysterectomy	Early in the postoperative period	Fever Frequency, urgency, dysuria	Suprapubic tenderness or tenderness to palpation of the anterior vaginal wall Urine culture: at least 100 000 colony‐forming units of a single organism that is not considered skin flora or at least 100 000 colony‐forming units on catheter specimen
Vaginal cuff cellulitis	Infection of the surgical margin in the upper vagina	0–8.3% after hys	Hysterectomy	Begin late in the hospital or usually after discharge	Pelvic pain Back pain Fever Abnormal vaginal discharge	PE: hyperemia, tenderness, induration of the vaginal cuff WBC: mildly to moderately ↑
Infected vaginal cuff hematoma or cuff abscess	Infection of the hematoma above the vaginal cuff	0–14.6% after hys	Hysterectomy	After discharge from the hospital, often later than the presentation of a cuff cellulitis	Fever Chills Pelvic pain Rectal pressure	PE: lower abdominal and vaginal cuff tenderness (often ↑ on one side), purulent drainage at the cuff, palpable fluctuant mass at the vaginal cuff Hb and Ht: ↓ US scan CT scan: delineate the hematoma
Postoperative pelvic abscess	Circumscribed collection of infected exudates	<1%	Hysterectomy, laparotomies, caesarian sections, D&C		High‐grade fever General malaise, nausea, vomiting, tachycardia, lower abdominal pain Vaginal discharge, vaginal bleeding, retention of urine Change in bowel habit	US scan and CT scan
Septic pelvic thrombophlebitis	Thrombosis in the pelvic veins	0.1%–0.5%	Gynecologic surgery	2–4 days after surgery	Fever, tachycardia, gastrointestinal distress Unilateral abdominal pain Palpable abdominal cord (acute thrombus formation)	PE: palpable abdominal cord resulting from acute thrombus formation (50%–67% of cases) CT scan, MRI
Pneumonia	Inflammatory condition of the lung primarily affecting the alveoli	0%–2.16% after hys	Hys, laparotomies	Early in the postoperative period	Shortness of breath, chills, chest pain, cough, and purulent sputum, fever	Examination may reveal decreased breath sounds, rales, hypoxia, tachycardia Chest radiograph can confirm and localize the infection, whereas sputum cultures can provide organism identification

Abbreviations: CT, computerized tomography; HB, hemoglobin; Ht, hematocrit; hys, hysterectomy; MRI, magnetic resonance imaging; PE, pelvic examination; US, ultrasound; WBC, white blood cell count.

Surgical site infections are an independent risk factor for postoperative sepsis in various surgical procedures. If not addressed promptly, SSIs can lead to complications such as fascial disruption or wound dehiscence, potentially progressing to necrotizing soft tissue infections and/or sepsis.^51^

Surgical site infections are classified into superficial incisional (skin or subcutaneous tissue), deep incisional (fascial and muscle layers), and organ/space infections (excluding the skin incision, fascial/muscle layers). These types of surgical infection are increasingly reported after hysterectomy.^52^

**FIGURE 1:**
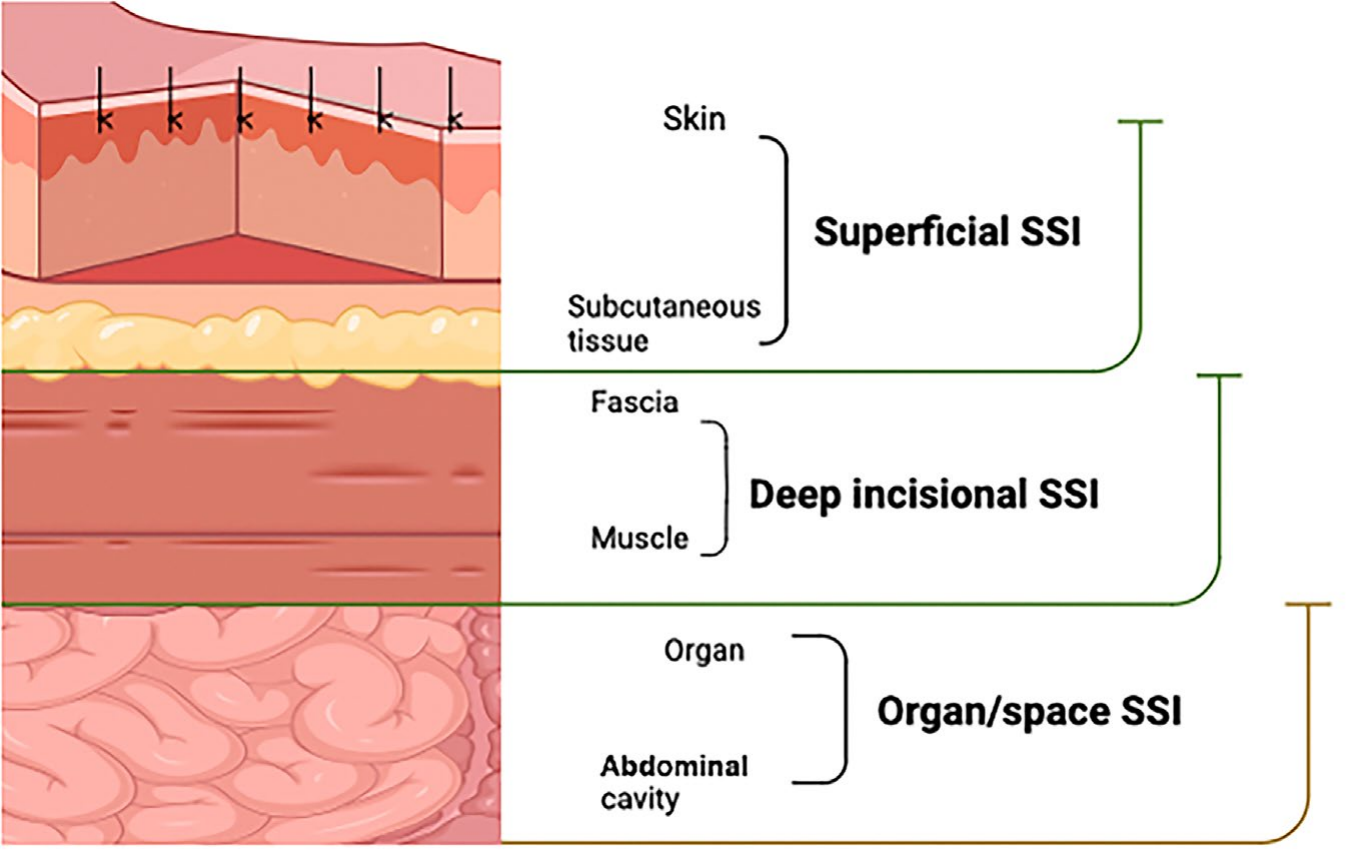
Surgical site infections.

The classification system proposed by the Centers for Disease Control and Prevention (CDC) devides surgical wound infections as follows:

- Superficial incisional SSI, which is primary (SIP) if it involves the primary incision, or secondary (SIS) if it involves the second incision. The most common superficial SSIs in gynecology are incisional cellulitis and vaginal cuff cellulitis. The cellulitis is frequently found as a complication of pelvic lymph node dissection surgery.
- Deep incisional SSI, which is primary (DIP) if it involves the primary incision in a patient who underwent an operation with one or more incisions, or secondary (DIS) if it involves the second incision. The most common deep incisional SSIs are deep tissue abscesses.
- Organ/space SSIs. These include tubo‐ovarian abscesses and pelvic abscesses, including vaginal cuff abscesses (Figure 1; Table 3).

**TABLE 3:** Classification of surgical site infection by the Centers for Disease Control (CDC).

	Definition	Criteria
Superficial incisional	Infection of skin or subcutaneous tissues within 30 d after surgery	One or more of the following: Purulent secretion of the superficial incision Isolation of organisms from a sample collected aseptically from the incision or the subcutaneous tissue Presence of at least one of the following: pain or tension, localized swelling, erythema or heat AND an incision deliberately opened by a surgeon or physician when a culture‐ or non‐culture‐based testing is not performed Diagnosis of superficial incisional SSI made directly by the surgeon or a physician
Deep incisional	Infection of deep soft tissues (fatty tissue, fascia or muscles) within 30 d after surgery or 90 d (if a prosthetic implant was used)	One or more of the following: Purulent drainage from the deep incision Spontaneous dehiscence of a deep incision or surgical revision AND identification of an organism AND one of the following: fever above 38°C, localized pain, or tenderness Presence of an abscess affecting the deep incision site detected by direct examination, reintervention, or radiological or histopathological examination
Organ/space SSI	Infection of the organs or spaces (under the fascia/muscle) manipulated during surgery within 30 d after surgery or 90 d (if a prosthetic implant was used)	One or more of the following: Purulent drainage from a drain placed in the organ/space correspondence Organism isolation from an aseptic fluid or tissue collection from the organ/space area Presence of an abscess affecting the organ/space detected by radiological examination or surgical revision

Abbreviation: SSI, surgical site infection.

In operative gynecology, the most frequent SSIs include vaginal cuff cellulitis, infected hematoma or abscess, and wound infection.

Vaginal cuff cellulitis refers to an infection at the surgical site in the upper vagina following a hysterectomy. Symptoms typically develop later in the postoperative period, even if the initial recovery was uneventful. On examination, findings may include persistent hyperemia, induration, tenderness of the vaginal cuff, and possibly purulent discharge and fever. The parametrial and adnexal areas remain non‐tender. Gram‐positive aerobes, facultative Gram‐negative aerobes, and obligate anaerobes can be associated with cuff cellulitis.^49^

Delayed complications, which often arise 10–14 days after surgery, can include an infected vaginal cuff hematoma, cuff abscess, or pelvic abscess. The latter is a rare complication of hysterectomy and may arise from the extension of pelvic cellulitis or an infected hematoma into the parametrial soft tissue. On physical examination and imaging, a fluctuant mass may be present, indicating a collection of infected fluid.^44^, ^49^

## 7 RISK OF INFECTION DURING GYNECOLOGICAL PROCEDURES

### 7.1 Hysteroscopy

The increased risk of infection associated with hysteroscopy can be attributed to several technical factors. As the vagina is naturally colonized by bacterial flora, the insertion of the hysteroscope through this area may facilitate the transfer of microorganisms to the upper genital tract. Additionally, the fluids used to distend the uterine cavity during the procedure can enhance the absorption of pathogens through the uterine walls, particularly if the tissue is traumatized during the procedure.^53^ Nevertheless, the risk of infection following hysteroscopy remains very low. In a prospective study including a total of 2116 hysteroscopies, only 30 infections (1.42%) were reported: 18 (0.85%) were endometritis and 12 (0.57%) were UTIs.^54^ A large Italian multicentric prospective study involving 42 934 hysteroscopies reported an infection rate of 0.06%.^55^

**FIGURE 2:**
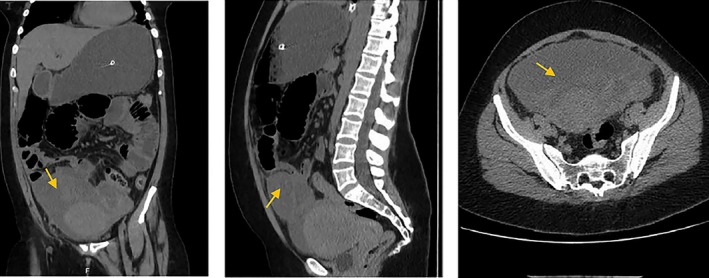
Rare case of ruptured tubal abscess and septic shock following hysteroscopy.

The most common infectious complications after hysteroscopy include endometritis, pyometras, and UTI. Patients with a history of pelvic inflammatory disease are at greater risk of developing post‐hysteroscopy infectious complications^56^ (Figure 2). For this reason, the presence of genital infection, including pyometra or active pelvic infection, are absolute contraindications to the procedure.^57^ In the Italian multicenter study mentioned earlier, among 25 cases of infection, five patients developed pelvic abscesses, all of whom had a history of severe endometriosis.^53^

Operative hysteroscopy carries a higher risk of infection compared with diagnostic hysteroscopy due to longer duration, greater invasiveness, and the repeated insertion and removal of the hysteroscope, increasing exposure to the vaginal flora. In a study comparing traditional hysteroscopy and vaginoscopy, no significant difference in the incidence of postoperative complications, including infections, was observed between the two groups.^58^

To date, there is no strong evidence supporting the use of antibiotic prophylaxis before hysteroscopy to prevent infections. A Cochrane review found no clear benefit of antibiotic prophylaxis before uterine transcervical procedures.^59^

Similarly, a RCT by Kasius et al., which included 365 women with infertility undergoing diagnostic hysteroscopy, showed no significant differences in infection rates between the antibiotic prophylactic and placebo groups.^60^ Comparable results have been reported in other RCTs focusing on operative hysteroscopy.^61^, ^62^

### 7.2 Laparoscopic and open abdominal myomectomy

When evaluating the incidence of postoperative infection after myomectomy and hysterectomy, it is crucial to clarify whether the definition of infection is based on infectious morbidity or febrile morbidity. Febrile morbidity is among the most common complications in both laparoscopic and laparotomic myomectomies and may result from infections, hematomas, or even unknown causes.

Although postoperative fever is significantly associated with longer hospital stays and the need for additional treatments, fever occurring within the first 24–48 h post‐surgery is commonly due to a systemic inflammatory response or cytokine release from tissue injury caused by the surgery.^63^ A recent study involving 249 women who underwent either abdominal (84.7%) or laparoscopic (15.3%) myomectomy found that approximately one‐third had febrile morbidity.^63^ BMI, abdominal approach, long operative time and postoperative anemia were significant independent risk factors for postoperative febrile morbidity. In this study, the overall incidence of febrile morbidity after myomectomy was 35.34%, including unexplained causes (91%), non‐surgical infections (8%), and SSIs (1%).^63^

A retrospective study by Rybak et al. compared 250 patients undergoing myomectomy with 341 patients undergoing hysterectomy and found similar rates of unexplained fever within the first 24 h. However, localized infectious complications (e.g., UTIs or pneumonia) were less common after myomectomy (14%) than after hysterectomy (31%).^64^

### 7.3 Hysterectomy

Hysterectomy is the most frequently performed major gynecological procedure, with approximately 600 000 hysterectomies performed annually in the United States.^65^ In a prospective nationwide study including a total of 10 110 hysterectomies, infections were the most common complications associated with hysterectomy for benign disease, ranging from 10.5% for abdominal hysterectomy to 13.0% for vaginal hysterectomy and 9.0% for laparoscopic hysterectomy.^66^

In a cross‐sectional analysis, Lake et al. reported that the overall incidence of cellulitis following hysterectomies was 1.6%, while only 0.03% of patients were diagnosed with both postoperative cellulitis and deep or organ‐space SSIs.^30^

Hysterectomy performed via an open approach is characterized by a higher risk of infections compared with minimally invasive surgery (MIS). In a retrospective study including 986 patients undergoing abdominal hysterectomy, the overall SSI rate following all hysterectomy procedures was 4.2%. More SSIs occurred in patients receiving the open technique (6.5%) than those who underwent laparoscopic (0%) or robotic (2.2%) procedures (*P* < 0.0001). Additionally, cases converted to open surgery also had an increased rate of SSIs (13.3%).^67^ Similarly, in another study, SSIs occurred more often after abdominal hysterectomy than after laparoscopic hysterectomy (4% vs. 2%, *P* < 0.01).^68^

A 2019 meta‐analysis including 176 016 patients found no significant difference in infectious complications rates between robotic‐assisted hysterectomy and laparoscopic‐assisted hysterectomy.^69^

Regarding laparoscopic entry techniques, a recent meta‐analysis reported no significant differences in the risk of trocar infection between direct trocar insertion and Veress needle insertion (OR 1.19, 95% CI 0.34–4.20, *P* = 0.78).^70^

Cellulitis rates after abdominal hysterectomy were 2.6%, compared with 0.6% in vaginal hysterectomy and laparoscopic hysterectomy, while infection rates in the deep/organ space were 1.2% in total abdominal hysterectomy, 1% in vaginal hysterectomy, and 0.5% in laparoscopic hysterectomy.^71^ Moreover, a Cochrane review concluded that laparoscopic and vaginal hysterectomy were associated with lower odds of febrile episodes, wounds, or abdominal wall infections compared with abdominal hysterectomy.^72^

## 8 DIAGNOSIS OF POST‐SURGICAL INFECTION

In a large retrospective analysis, Wright et al. found that among patients with infectious complications following major gynecologic surgery, the rate of failure to rescue—defined as death that occurs after a potentially treatable postoperative complication—increased from 0% in the hospitals with the lowest mortality rates to 8.1% at the hospitals with the highest mortality rates.^20^ Specifically, mortality rates were associated with failure to rescue but not with the complications rate itself. Therefore, prompt diagnosis of postoperative infection is crucial.

Patients with SSIs often present with pain and tenderness at the operative site, and fever. However, several studies have shown that fever is not a specific indicator of infection.^73^, ^74^, ^75^ In a large study of 537 patients undergoing major gynecologic surgery, Fanning et al. reported that 39% developed postoperative fever, but a documented infection was found in only 8% of these cases.^76^ Similarly, the multicenter CREST study of 1283 abdominal hysterectomy patients found a 32.3% rate of febrile morbidity among postoperative hysterectomy patients. In this study, the febrile morbidity rate was approximately twice as high in the abdominal hysterectomy group than in the vaginal hysterectomy group, although the distribution of fever causes was similar across both groups. Additionally, no identifiable source of infection was found in about 50% of patients with fever, regardless of the surgical approach.^77^

Thus, a thorough history and physical examination are crucial in guiding imaging and laboratory evaluation. Clinical signs such as skin erythema, subcutaneous induration, and/or spontaneous drainage of serous or purulent fluid are usually observed. Pelvic examination may reveal vaginal cuff, paravaginal, or pelvic organ tenderness. Wound swabbing, culture, and laboratory tests are used to isolate the microorganism and to assess leucocyte count. Imaging modalities such as ultrasound, magnetic resonance imaging, or computed tomography scan may be employed to localize the infection site.^22^

Some molecular mediators of sepsis can be used as biomarkers for early diagnosis and prognosis. In clinical practice, the most commonly used biomarkers include white blood cell (WBC) count, C‐reactive protein (CRP), procalcitonin (PCT), and lactate levels.

White blood cell count is widely used as a marker of bacterial infections and to monitor response to antibiotic therapy.^22^

- CRP is an acute‐phase cytokine produced by the liver via upregulation of IL‐6. CRP plays a key role in mediating acute inflammation, representing an indicator of significant inflammatory or infectious disease, either alone or in combination with WBC. CRP levels rise within a few hours of activation, peaking within 48 h post‐infection. The CRP/albumin ratio, rather than CRP levels alone, can serve as a risk factor or predictor of 90‐day mortality in septic patients and as a long‐term prognostic biomarker.^22^
- PCT is the precursor of calcitonin, which regulates blood calcium levels. PCT has been found to be more specific for bacterial infection than CRP, particularly in surgical patients postoperatively. PCT has a rapid induction time (3–4 h) and its levels decrease with antibiotic treatment, making it useful to guide decisions on the discontinuation of antibiotic treatment.^78^
- Lactate is constantly produced by glycolysis in red blood cells and tissues, and is converted to glucose by the liver. Elevated lactate levels may indicate organ dysfunction or damage due to increased glycolysis or impaired liver dysfunction. Levels above 4.0 mmol/L are associated with multiorgan damage and worse prognosis.^24^

In 2016, the international consensus redefined sepsis, introducing the Sequential Organ Failure Assessment (SOFA) and quick SOFA (qSOFA) score to assess organ dysfunction objectively. According to this definition, sepsis is now defined as the presence of an infection combined with an acute change in SOFA or qSOFA score—by over 14% or at least 2 points, respectively.^6^

The SOFA scoring system evaluates functional impairment of six systems—respiratory, cardiovascular, coagulation, renal, hepatic, and neurological—and predicts mortality risk: a score of 0–6 corresponds to <10% mortality; scores of 13–14 correspond to approximately 50% mortality; and for scores >15 the expected mortality is 90%.^79^

The qSOFA criteria include three criteria: systolic blood pressure of 100 mmHg or less, respiratory rate of 22/min or greater, and altered neurological status. If a patient meets two or more of these criteria, it suggests a higher likelihood of sepsis and the need for urgent evaluation and intervention.^79^

## 9 SSI REDUCTION BUNDLES

Prophylactic antibiotics have been shown to reduce infectious morbidity in most major gynecological surgeries, including hysterectomy. For antibiotic prophylaxis to be effective, several key conditions must be met: (1) the surgical procedure should carry a significant risk of bacterial contamination; (2) the prophylactic antibiotic should target the expected pathogens and have minimal side effects; (3) it should not be an antibiotic typically used for therapeutic treatment; and (4) the antibiotic must reach optimal tissue concentrations at the time of surgery.

International guidelines recommend antibiotic prophylaxis before invasive gynecological interventions such as vaginal hysterectomy, abdominal hysterectomy, and cesarean section.^80^, ^81^

The 2019 ERAS guidelines in gynecologic oncology added recommendations for surgical site infection reduction bundles such as antimicrobial prophylaxis, skin preparation, avoiding hypothermia, avoiding surgical drains, and control of perioperative hyperglycemia.

First‐generation cephalosporins should be administered within 1 h before the surgical incision, with dosing adjusted based on the patient's weight. Antibiotic regimens should be tailored to the planned procedure, with the addition of anaerobic coverage added for pelvic cancer surgery or bowel surgery. In addition, prophylactic administration of 500 mg of intravenous metronidazole before and 8 h after radical hysterectomy reduces the risk of post‐surgical infections (12% in the placebo group vs. 6% in the antibiotic group).^82^ Skin preparation is another key element in reducing SSIs. Patients should shower before surgery with a chlorhexidine‐based antimicrobial soap and undergo a chlorohexidine‐alcohol skin preparation in the operating room. Additionally, hypothermia should be avoided, as it is associated with increased SSI risk and cardiac events. The use of surgical drains (peritoneal, subcutaneous, and nasogastric drains) should generally be avoided after abdominal surgery, although high‐quality evidence supporting this recommendation is lacking. Reducing perioperative hyperglycemia is also important; glycemic levels should be kept under 200 mg/dL in diabetics and non‐diabetics, which should be screened.

Finally, preoperative cleaning of the hands is essential for preventing SSIs. This involves removing jewelry and watches, and washing hands with an alcoholic solution or antimicrobial soaps and the forearms with an antiseptic agent. Cleaning with an alcohol‐based aqueous solution can be as effective as traditional handwashing with antiseptic soap for the prevention of SSIs. The recommended duration of rubbing with alcohol‐based hand products is about 60 s. Hand hygiene must be practiced by all members of the surgical team.

In 2024, a study demonstrated that implementation of an SSI bundle within an ERAS care pathway was associated with a reduction in SSIs and infectious complications in gynecologic oncology surgery.^10^ Furthermore, cost‐effectiveness was reported in several studies of ERAS in gynecologic oncology with statistically significant cost savings.^83^

## 10 CONCLUSIONS

Infectious complications following gynecological surgery increase healthcare costs and lead to prolonged hospital. Understanding risk factors, types of infection and prevention strategies according to the updated ERAS recommendation could help gynecological surgeons in preventing sepsis and failure to rescue events.

## AUTHOR CONTRIBUTIONS

Concept and design: CT, PG, CM, MG, FN, RM; acquisition, analysis, or interpretation of data: CT, MG, CM, FN, MA; drafting of the manuscript: CT, CM, MG, FN; critical revision of the manuscript for important intellectual content: GS, GB, OB, PG, GV, NB, MP; administrative, technical, or material support: CT; supervision: PG, GS, GV, NB, GB, SR.

## FUNDING INFORMATION

TC is supported by the Department of Medical Sciences, University of Ferrara, through a grant awarded under the ‘5x1000 Young Researchers 2025’ funding program.

## CONFLICT OF INTEREST STATEMENT

The authors have no conflicts of interest.

## Data Availability

Data sharing is not applicable to this article as no new data were created or analyzed in this study.
